# Phylogenomic incongruence in *Ceratocystis*: a clue to speciation?

**DOI:** 10.1186/s12864-020-6772-0

**Published:** 2020-05-14

**Authors:** Aquillah M. Kanzi, Conrad Trollip, Michael J. Wingfield, Irene Barnes, Magriet A. Van der Nest, Brenda D. Wingfield

**Affiliations:** 1 Department of Biochemistry, Genetics and Microbiology, Forestry and Agricultural Biotechnology Institute, University of Pretoria, Pretoria, South Africa; 2 School of Applied Systems Biology, La Trobe University, Bundoora, Victoria Australia; 3 Agriculture Victoria Research, Department of Jobs, Precincts and Regions, AgriBio Centre, Bundoora, Australia; 4 Biotechnology Platform, Agricultural Research Council, Onderstepoort Campus, Pretoria, South Africa

**Keywords:** *Ceratocystis*, Incongruence, Hybridisation, Phylogenomics

## Abstract

**Background:**

The taxonomic history of *Ceratocystis,* a genus in the Ceratocystidaceae, has been beset with questions and debate. This is due to many of the commonly used species recognition concepts (e.g., morphological and biological species concepts) providing different bases for interpretation of taxonomic boundaries. Species delineation in *Ceratocystis* primarily relied on genealogical concordance phylogenetic species recognition (GCPSR) using multiple standard molecular markers.

**Results:**

Questions have arisen regarding the utility of these markers e.g., ITS, BT and TEF1-α due to evidence of intragenomic variation in the ITS, as well as genealogical incongruence, especially for isolates residing in a group referred to as the Latin-American clade (LAC) of the species. This study applied a phylogenomics approach to investigate the extent of phylogenetic incongruence in *Ceratocystis*. Phylogenomic analyses of a total of 1121 shared BUSCO genes revealed widespread incongruence within *Ceratocystis*, particularly within the LAC, which was typified by three equally represented topologies. Comparative analyses of the individual gene trees revealed evolutionary patterns indicative of hybridization. The maximum likelihood phylogenetic tree generated from the concatenated dataset comprised of 1069 shared BUSCO genes provided improved phylogenetic resolution suggesting the need for multiple gene markers in the phylogeny of *Ceratocystis.*

**Conclusion:**

The incongruence observed among single gene phylogenies in this study call into question the utility of single or a few molecular markers for species delineation. Although this study provides evidence of interspecific hybridization, the role of hybridization as the source of discordance will require further research because the results could also be explained by high levels of shared ancestral polymorphism in this recently diverged lineage. This study also highlights the utility of BUSCO genes as a set of multiple orthologous genes for phylogenomic studies.

## Background

Delineation of species boundaries is a complex and highly contentious topic among evolutionary biologists. Ideally, a species should be defined as representing a single lineage that maintains its identity from others, with its own evolutionary tendencies and historical fate [1]. In fungi, species recognition is generally based on three commonly applied concepts i.e., the Biological Species Concept (BSC), the Morphological Species Concept (MSC) and the Phylogenetic Species Concept (PSC) [2, 3]. Typically, species are recognised based on the application of systematic characters to reliably distinguish all individuals belonging to a defined group or lineage. MSC and BSC are trait-based and species are grouped using visibly measurable traits such as morphology or reproductive compatibility [4]. PSC differs from MSC and BSC in that it makes use of conservation in DNA sequences to represent shared ancestry [4].

Species delineation is the taxonomic practice that is used to describe an organism in relation to others [5]. Species boundaries defined using BSC, PSC and MSC in fungal systematics are challenged by recurrent inconsistencies [4, 5]. For example, in the case of the BSC and MSC, species numbers could be underestimated due to the extended time periods for changes in morphology or mating compatibility to become evident [2]. PSC determines species boundaries objectively by measuring DNA changes over time [6]. As such, it could be argued that the PSC offers the best possible approach because changes in gene sequences can be easily related to evolutionary time. Trait-based concepts typically lead to ambiguous outcomes due to convergent evolution of morphological traits and where cryptic species are commonly overlooked [2]. In this regard, cryptic speciation is common in, but not limited to, groups that comprise large numbers of species such as the prokaryotes and fungi [5].

*Ceratocystis* is one of numerous genera that reside in the family Ceratocystidaceae, order Microascales, and class Sordariomycetes [7]. Species in this family include important plant pathogens that cause serious disease, both in agricultural crops and in natural ecosystems [8–10]. Application of the PSC for *Ceratocystis* reveals four geographically defined groups. These include the North American clade (NAC) [11], the Latin American clade (LAC) [12, 13], the African clade (AFC) [14, 15] and the Asian-Australian clade (AAC) [11, 16, 17]. Yet problems regarding the taxonomy of *Ceratocystis* remain prominent. For example, Fourie et al. [18] were not able to distinguish between *C. manginecans* and *C. acaciivora* using commonly used molecular markers and reduced these species to synonymy. Similarly, Oliveira et al. [19] could not distinguish among phylogenetic lineages of *C. manginecans, C. eucalypticola* and *C. fimbriata* using BSC and consequently regarded these three species as a single taxon represented by multiple distinct genotypes. Other researchers (Harrington et al. and Li et al. [20, 21]) suggest that isolates of *C. fimbriata, C. manginecans* and *C. eucalypticola* represent a single South American species that has been introduced on different hosts to other continents by humans.

The Internal Transcribed Spacer (ITS) region of ribosomal RNA genes is generally treated as the barcode region used for fungal species identification [22]. It is often used in combination with additional gene regions such as β-tubulin and translation elongation factor 1-α to delineate species utilising Genealogical Concordance Phylogenetic Species Recognition (GCPSR) [2]. But the ITS region, especially when it is used alone, is not considered reliable for species delineation in *Ceratocystis* [18, 19]. This is due to intragenomic variation of multiple ITS gene within individual isolates of *Ceratocystis* [23, 24]. This variation was initially observed in a single *C. manginecans* isolate (LAC), which included ITS types similar to the ITS of two distinct species [23, 25, 26] but many other examples have arisen more recently [27, 28].

Intragenomic variation in the ITS region has been associated with hybridization [29, 30]. Ribosomal genes occur as tandem repeats and the intragenomic copies, or paralogs, are usually conserved due to concerted evolution [31]. The mechanisms responsible for this phenomenon include gene conversion and unequal crossing over [32]. In plants, hybridization leads to the retention of both parental ITS types, homogenization to a single ITS sequence and/or homogenization of elements of each parental ITS type into a single composite sequence [29]. Hybridization was first suggested to occur in *Ceratocystis* by Engelbrecht and Harrington [12]. A study on *Ceratocystis manginecans* to elucidate the causes of intragenomic variation in the ITS region demonstrated the effects of unequal crossing over, and potentially gene conversion, to explain the random homogenization toward a specific ITS type in culture [23]. The results suggested that the observed polymorphisms in the ITS region could have originated from a hybridization event.

Phylogenetic incongruence in *Ceratocystis,* and the presence of multiple ITS types within individual isolates has raised many questions regarding species boundaries in this genus. Phylogenomic analyses have been used to resolve incongruent phylogenetic relationships [33], analyse incongruence of genes and their histories, understand population dynamics and to explore evolutionary patterns acting across the genome [34]. The aim of this study was to use a phylogenomic approach to (i) identify a set of orthologous genes shared across the Ceratocystidaceae (ii) use these genes to identify the extent of discordance among gene trees, (iii) and analyse the alternative topologies within *Ceratocystis*, specifically within the LAC. The overall objective was to explore the possible role of hybridization and/or introgression that might explain phylogenetic discordance in the group. This approach allowed for a comprehensive species tree estimation using GCPSR with the largest dataset used thus far for this genus. This phylogenomic study made use of the Benchmarking Universal Single-Copy Orthologs tool [BUSCO] method [35] as the basis for ortholog selection.

## Results

### Genome information

The genomes and genome assembly statistics are summarised in Table 1. Genome sizes in *Ceratocystis* varied between 27 to 30 Mb. These genomes were of high quality, as shown by their N50 values (Table 1) and genome completeness based on BUSCO analyses (Table 2). The representative isolates have a broad geographical distribution, including North America, Africa, Europe and South East Asia.

**Table 1:** General information and assembly statistics of the 17 Ceratocystidaceae isolates used in this study

Species	Isolate number/Strain	Code^**a**^	Country	Host (Genus)	Genome accession number	Size (Mb)	N50	Contigs^**b**^ (> 1 kb)
*C. albifundus*	CMW17620	CALB1	South Africa	*Terminalia*	JSSU00000000	27.15	58,335	939
	CMW4068	CALB2	South Africa	*Acacia*	MAOA02000000	27.32	50,568	1003
	CMW17274	CALB4	South Africa	*Faurea*	MANX00000000	26.56	22,532	2122
	CMW24685	CALB5	Kenya	*Acacia*	MANZ00000000	27.12	48,054	1072
	CMW24860	CALB6	Tanzania	*Acacia*	JAAUVK000000000	27.42	62,926	1064
*C. eucalypticola*	CMW9998	CEUC	South Africa	*Eucalyptus*	LJOA00000000	31.26	116,489	961
*C. fimbriata*	CMW15049	CFIM1	USA	*Ipomoea*	JAAVJK000000000	29.5	13,763	3545
	CMW14799	CFIM2	USA	*Ipomoea*	APWK00000000	29.5	174,236	399*
*C. harringtonii*	CMW14789	CHAR	Poland	*Populus*	MKGM00000000	31.06	66,000	813*
*C. manginecans*	CMW17570	CMAN1	Oman	*Prosopis*	JJRZ00000000	31.71	77,070	980*
	CMW22563	CMAN2	Indonesia	*Acacia*	VIFZ00000000	31.87	606,428	231*
	CMW46461	CMAN3	Malaysia	*Acacia*	SGIO00000000	31.8	598,724	225*
*C. platani*	CFO	CPLA	Italy	*Platanus*	LBBL00000000	29.18	77,580	1213
*C. smalleyi*	CMW14800	CSMA	USA	*Carya*	NETT00000000	27.3	–	1242
*D. virescens*	CMW17339	DVIR	USA	*Acer*	LJZU00000000	33.65	118,189	561
*E. polonica*	CMW20930	EPOL	Norway	*Picea*	LXKZ00000000	32.46	86,326	914*
*E. laricicola*	CMW20928	ELAR	Scotland	*Larix*	LXGT00000000	32.79	77,789	879*

^a^Species code used in this study for identification of each isolate. The first letter represents the genus, while the following three letters correspond to species name. Numbers at the end of codes represent different isolates of the same species

^b^Number of contigs greater than 500 bp

**Table 2:** The genome completeness score assessed by BUSCO on all Ceratocystidaceae genomes

Species name	Code	BUSCO notation (%)				Complete SCG^**a**^	Complete DG^**b**^	Fragmented	Missing
		Completed	Duplicated	Fragmented	Missing				
*C. albifundus*	CALB1	97	7.5	1.5	1	1400	109	23	15
	CALB2	97	7.5	1.1	1.4	1401	108	16	21
	CALB4	97	7.8	1.3	1.2	1401	113	19	18
	CALB5	97	7.6	1.2	1.2	1402	110	18	18
	CALB6	97	11	1.2	1.2	1402	172	18	18
*C. eucalypticola*	CEUC	98	7.5	0.6	1	1413	108	10	15
*C. fimbriata*	CFIM1	97	7.4	0.7	1.4	1406	107	11	21
	CFIM2	98	7.5	0.4	1	1416	109	7	15
*C. harringtonii*	CHAR	98	7.1	0.9	0.9	1410	103	14	14
*C. manginecans*	CMAN1	97	7.8	1.2	0.9	1407	113	18	13
	CMAN2	97	7.5	1	1	1408	109	15	15
	CMAN3	98	7	0.8	0.8	1414	101	12	12
*C. platani*	CPLA	98	7.9	0.8	1.1	1410	115	12	16
*C. smalleyi*	CSMA	98	12	0.9	0.7	1413	182	14	11
*D. virescens*	DVIR	98	6.5	0.9	0.4	1418	94	13	7
*E. polonica*	EPOL	98	6.7	0.6	0.9	1415	97	10	13
*E. laricicola*	ELAR	98	7.3	0.5	0.9	1417	105	8	13

^a^The number of Complete Single-Copy Genes

^b^The number of Complete Duplicated Genes

### Ortholog selection using BUSCO analysis

BUSCO analysis of the 17 Ceratocystidaceae genomes showed high levels of completeness (Table 2) with scores between 97 and 98%. An average of 1409 complete, single-copy BUSCO genes were successfully identified across all genomes. The average number of duplicated BUSCOs was approximately 7.5%, with all genomes showing little fragmentation and low levels of missing genes (± 1%). Orthologs for phylogenomic analysis were selected based on BUSCO genes that were complete, and present in single copy in each genome. A total of 1123 BUSCOs were found to be shared within *Ceratocystis*. Of these, 1121 BUSCO sequences were retained after curation and considered for phylogenomic analysis. When the outgroup taxa *Davidsoniella* and *Endoconidiophora* were used, the total was 1082 BUSCOs with 1069 nucleotide alignments being retained after curation.

### Phylogenetic analyses

**Fig. 1:**
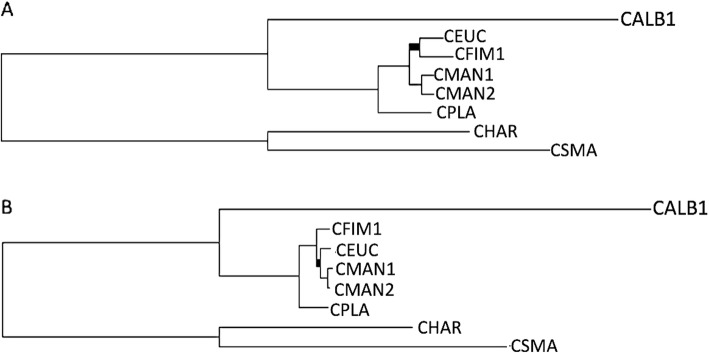
Maximum likelihood (ML) species tree estimates of *Ceratocystis* species using concatenated datasets of both amino acid (**a**) and nucleotide (**b**) sequences. All nodes are supported by 100% bootstrap values (not shown). Thickened branches represent difference in topology between the 2 ML species trees using the Pairwise comparison software Compare2trees (Nye et al. [36])

Functional annotation of the 1082 complete BUSCOs revealed that these genes were predominantly associated with primary cellular functions, including cellular regulation, organization and related key processes (Additional file 1: Figure S1). To determine the phylogenetic relatedness of *Ceratocystis* spp., initial analyses only included *C. smalleyi*, *C. manginecans*, *C. albifundus*, *C. platani*, *C. fimbriata*, and *C. eucalypticola*. Two maximum likelihood (ML) species trees were generated using curated concatenated amino acid sequence alignments (633,499 aa) and nucleotide alignments (approximately 2.2 Mbp long). These data were obtained from a total of 1121 shared BUSCO genes. The species tree nodes were well supported with bootstrap values of 100% observed in all nodes (Fig. 1). Incongruence between the amino acid and nucleotide ML species tree topologies was observed between *C. manginecans*, *C. fimbriata* and *C. eucalypticola*. The amino acid ML species tree placed *C. fimbriata* and *C. eucalypticola* as a sister clade to *C. manginecans* (Fig. 1a). In contrast, the nucleotide ML species tree placed *C. eucalypticola* and *C. manginecans* as a clade separate from *C. fimbriata* (Fig. 1b).

**Fig. 2:**
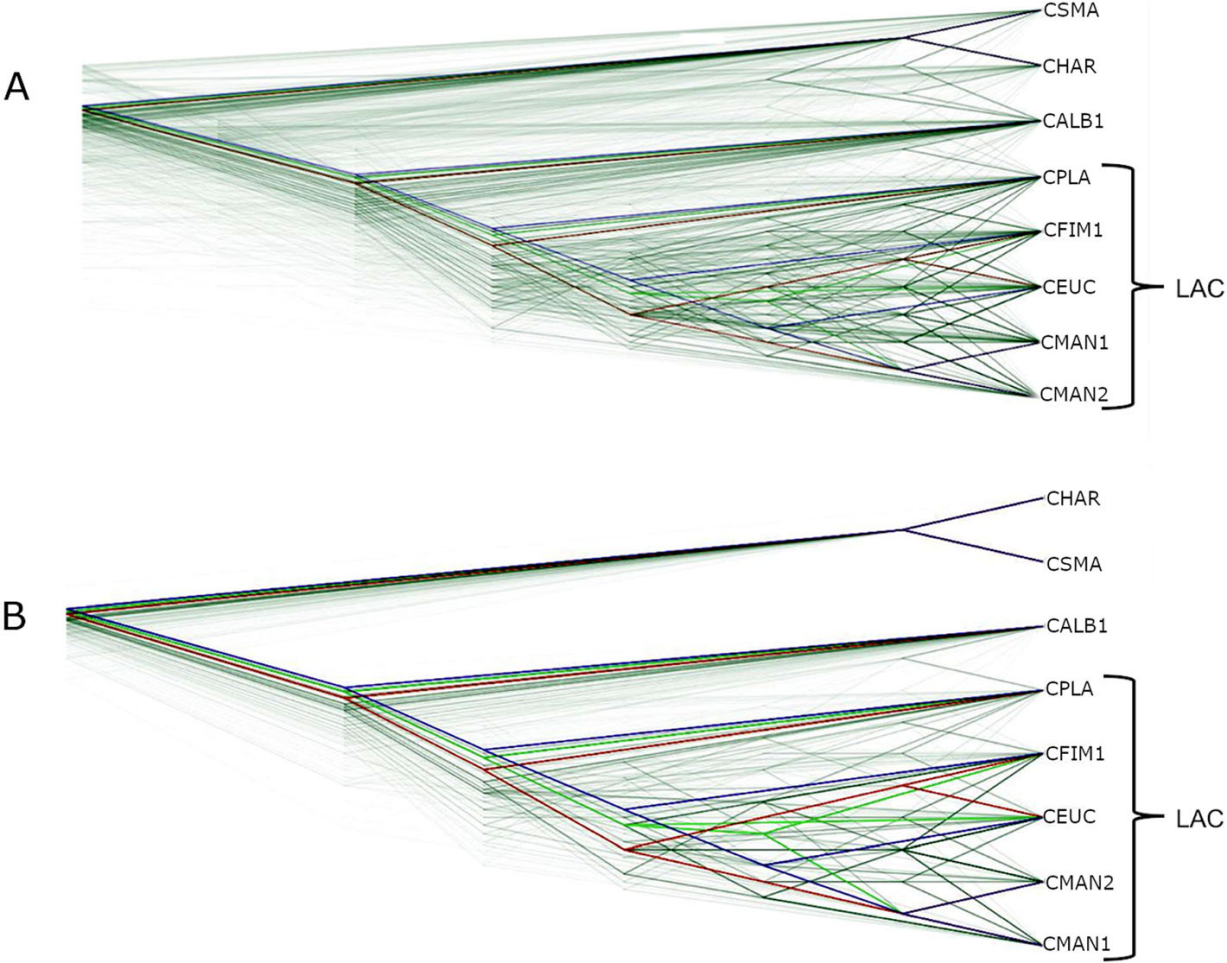
DensiTree analysis of 1121 amino acid and nucleotide ML gene trees of *Ceratocystis* species. DensiTree analysis revealed 448 and 99 different topologies in the amino acid (**a**) and nucleotide (**b**) maximum likelihood (ML) trees respectively drawn using default tree drawing parameters. Consensus trees coloured red, bright green and blue represent the three most supported topologies

Further analysis of incongruence among the 1121 amino acid ML tree set using DensiTree revealed 448 consensus tree topologies present in the tree set (Fig. 2a). Tree topologies showed incongruent branches throughout the dataset, including inconsistencies in the deeper nodes of the tree. MetaTree analysis showed a star-like pattern, with support for four consensus nodes (Additional file 2: Figure S2 A). Although not a complete representation of the number of gene trees supporting each topology, the star-tree like pattern illustrated the major incongruence of this dataset. Topologies represented by the four consensus nodes lacked phylogenetic resolution and did not resolve the species relationships. None of the consensus trees resolved *C. platani* as a distinct lineage, while the two smaller consensus trees either lacked resolution for *C. albifundus* or showed no resolution across the analysed *Ceratocystis* spp.

DensiTree analysis of the nucleotide 1121 gene ML tree set showed a reduction in the number of alternative topologies (99) compared to the amino acid dataset (448). Discordance patterns were mostly observed within the *C. manginecans, C. fimbriata* and *C. eucalypticola* clade (Fig. 2b). Approximately 73% of the gene trees show incongruence occurring within *C. fimbriata*, *C. manginecans* and *C. eucalypticola*. Despite some incongruence involving *C. platani* and to a lesser extent *C. albifundus* (CMW17620), the dataset supported the distinction of these species from *C. manginecans* and *C. fimbriata*. Three main topological patterns were evident within the *C. manginecans* and *C. fimbriata* lineage (Fig. 2b and Additional file 3: Figure S3). These topologies were supported by approximately 17% of the ML gene trees. DensiTree analysis further showed that clade probability levels within this group range between 21 and 32%, with the larger percentage supporting the grouping of *C. eucalypticola* with *C. manginecans*. MetaTree analysis again revealed a star-like topology, but the improved resolution using nucleotide data revealed a greater number of tree clusters (Additional file 2: Figure S2 B). Although most the consensus trees included *C. platani* as a part of the incongruent clade, the proportions of support for these consensus trees was masked by other topologies.

**Fig. 3:**
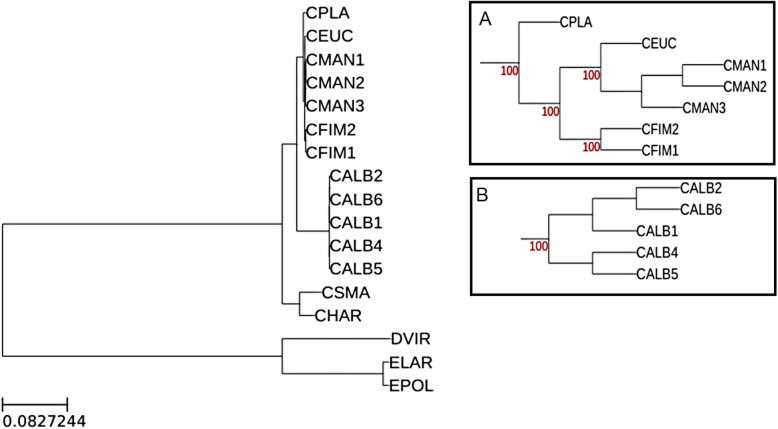
Maximum likelihood species phylogeny of the 17 Ceratocystidaceae isolates used in this study. The parameters used in the ML include the GTRGAMMA model of evolution and 1000 bootstrap replicates for branch support estimation. All nodes supporting each species are supported by 100% bootstrap values. Bootstrap for nodes supporting isolates of the same species were below 100% as expected (not shown). Insets A and B are zoomed in images of the *C. manginecans* and *C. albifundus* clades respectively

To better understand the levels of incongruence seen in the *C. manginecans*, *C. eucalypticola* and *C. fimbriata* clade, an expanded dataset including 5 *C. albifundus* isolates was analysed. These were specifically used to compare the patterns of incongruence within a well-defined species [37, 38]. In addition, outgroups (*D. virescens*, *E. polonica* and *E. laricicola*) were included to root the phylogenetic trees. The final dataset included 17 Ceratocystidaceae isolates used in this study (Table 1). After concatenation and curation of the 1082 BUSCO genes shared among the expanded dataset, we inspected the alignment and removed genes that were not present in all 17 isolates leaving 1069 BUSCO genes. For this analysis only nucleotide data were considered due to the low signal caused by widespread conservation in the amino acid sequences in the initial analysis including only *Ceratocystis* species. The ML and Bayesian species tree estimation was performed using a concatenated dataset (again approximately 2 Mbp long) including all 1069 shared BUSCO sequences. Both ML and Bayesian species trees showed separation between *C. manginecans* and *C. eucalypticola* supporting previous findings [7] (Fig. 3 and Additional file 4). The branch lengths in the *C. manginecans* lineage were short however, there was evidence to suggest a deeper branching pattern compared to the *C. albifundus* lineage (Fig. 3).

**Fig. 4:**
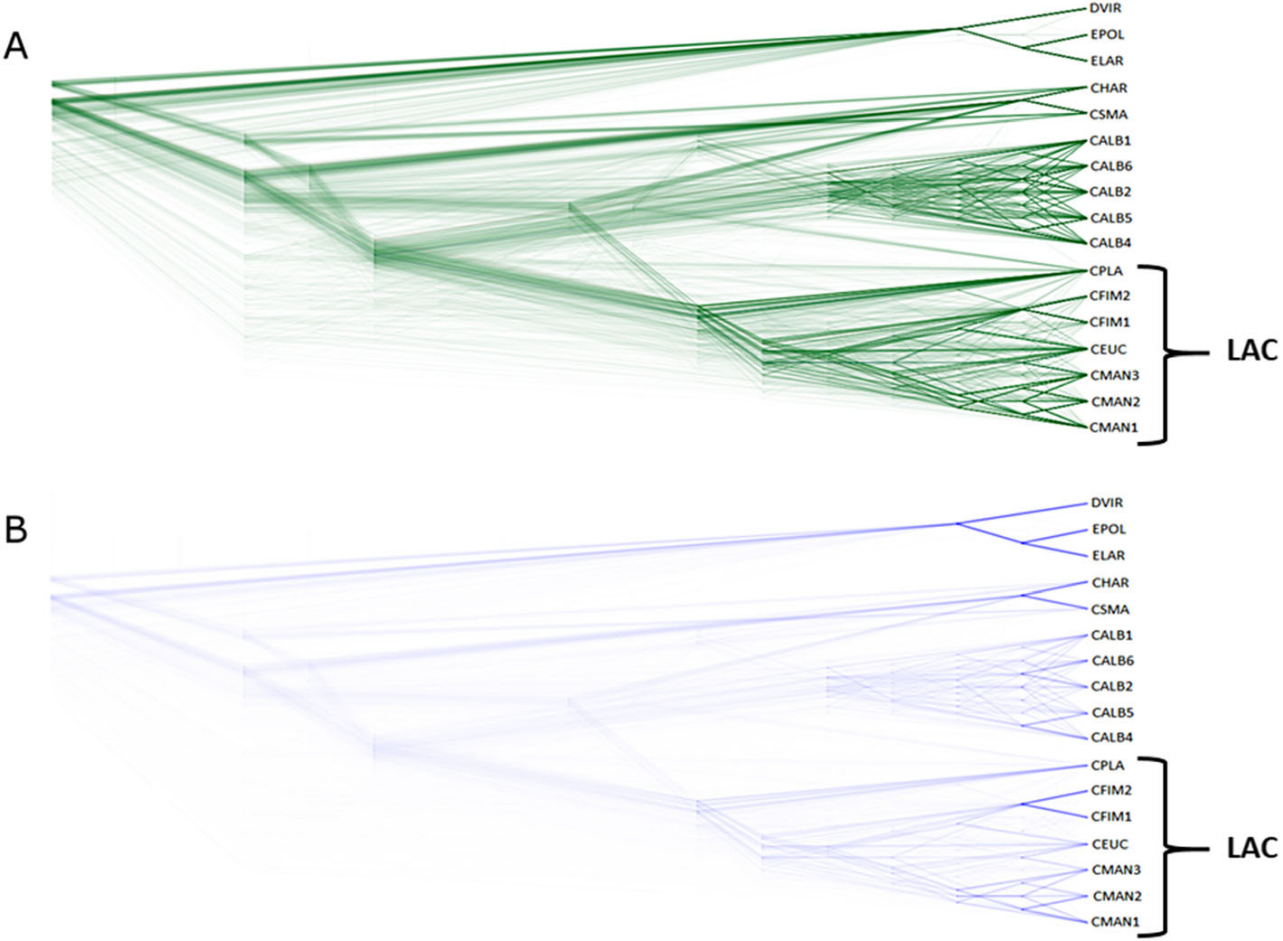
DensiTree analysis of phylogenetic trees of 1069 concatenated gene sequences including all 17 isolates analysed in this study. This image illustrates the difference in branching patterns between the well-defined lineage of CALB (*C. albifundus*) and the more divergent groupings of CEUC-CMAN (*C. eucalypticola* and *C. manginecans*) and CFIM (*C. fimbriata*). **a** – DensiTree image of all trees drawn with default drawing settings using the ‘Closest First’ Shuffle. **b** – DensiTree image of the consensus tree topologies drawn using the star-tree drawing option to illustrate branching patterns of the ML phylogenies. LAC denotes Latin American Clade

Incongruence analysis of the nucleotide ML gene tree set of 1069 concatenated BUSCOs shared among the 17 Ceratocystidaceae genomes analysed using DensiTree revealed 977 consensus tree topologies (Fig. 4a and b). There were several incongruent branches deep within the tree space, showing uncertainty in the divergence patterns of *Ceratocystis*. The deep branching pattern of the LAC was distinct, but a less uniform pattern was observed towards the terminal nodes. This was especially true for *C. eucalypticola* where a less uniform pattern with no clear branching point was observed. In contrast, the divergence of the *C. fimbriata* and *C. manginecans* was clear.

## Discussion

Several species concepts have recently been applied to determine species boundaries in *Ceratocystis* [18, 19]. Species concepts in the phylogenetics era are however, constantly being challenged. This is particularly true when the regions/markers applied have conflicting signals due to lack of resolution, as seen for highly conserved genes or where there are high levels of ancestral polymorphism. The results of this study call to question the utility of employing small numbers of molecular markers when defining species boundaries.

The ML phylogenetic tree generated using the concatenated nucleotide dataset covering 17 genomes and seven species in this genus and over 1000 loci support the phylogenetic relationships established by the recent taxonomic study for alternative markers in *Ceratocystis* [18]. Previous studies have failed to differentiate between *C. manginecans*, *C. eucalypticola* and *C. fimbriata* isolates using BSC [19] but the ML phylogenetic tree placed *C. fimbriata* as a separate lineage from *C. manginecans* and *C. eucalypticola*. Results of the present study also suggest that BUSCOs [35], can be helpful in resolving taxonomic questions such as those for *Ceratocystis*, where commonly used nuclear markers fail to delineate species. Indeed, these BUSCO genes could complement previous efforts to identify molecular markers for delineating *Ceratocystis* species [18].

ML phylogenies obtained from nucleotide and amino acid datasets revealed incongruence in *Ceratocystis*. For example, discordance between the species tree topologies was observed among *C. manginecans*, *C. eucalypticola* and *C. fimbriata*. While the amino acid ML phylogenetic tree placed *C. fimbriata* and *C. eucalypticola* as a sister clade to *C. manginecans*, the nucleotide ML species tree placed *C. eucalypticola* and *C. manginecans* as a clade separated from *C. fimbriata*. Similar incongruence was observed between individual nucleotide and amino acid ML gene trees. The results of this study emphasise the importance of analysing a dataset comprised of multiple genes for species delineation [39]. This is particularly relevant for species of *Ceratocystis* residing in the LAC where the branching pattern is difficult to determine.

The hypothesis that *Ceratocystis* is a recently diverged lineage was raised in a recent study of Van der Nest et al. [40] where the age of speciation events in the Ceratocystidaceae was estimated. Short branch lengths separating these lineages as shown by the ML species phylogeny for *Ceratocystis* especially within the LAC, and the patterns of incongruence observed in this study are characteristics of recently diverged lineages [41]. Notwithstanding our findings, the possibility that the incongruence patterns in *Ceratocystis* are due to the use of highly conserved genes cannot be excluded. The resolution offered by the BUSCOs, which provide a large sample size of conserved orthologs present in all fungi [35], may not be sufficient, thus complicating the process of species delineation. As a case in point, in our study we were not able to resolve *C. platani* as a distinct lineage despite using more than 1000 gene loci.

Introgressive hybridisation or shared ancestral polymorphism are the most common biological causes of phylogenetic tree incongruence [42]. Both factors manifest in the same way when assessing tree topologies. There is no reliable way to distinguish between these possibilities, although several have been proposed [43, 44]. The results of the present study show incongruence patterns in the LAC group of *Ceratocystis*, which may be expected in lineages that have undergone introgression. Introgression, or gene flow, is also most common in populations that constantly undergo admixture, or in populations that are in the process of divergence [6]. In a study by Lee et al. [45], an intermediate level of gene flow was reported in populations of *C. albifundus*. Overall, the results of the present study appear to reflect a situation in *Ceratocystis* where speciation is occurring and where gene flow will continue until barriers are established through absolute divergence [6].

Closely related species of *Ceratocystis* such as those related to *C. fimbriata* display a high level of host specificity. For example, the sweet potato pathogen that defines the genus infects only this host and isolates represent a single globally distributed clone that has recently been designated as a *forma specialis* of *C. fimbriata* [46]. Other species such as *C. manginecans* that also display relatively limited genetic variability have a much wider host range that could have been caused by undetected positive selection. How these should be treated taxonomically has yet to be resolved but this clearly requires an analysis of large populations of isolates, from different hosts and geographic locations. In this regard, species of *Ceratocystis* provide a useful example to explore species concepts in a fungal lineage that is currently undergoing divergence.

A phylogenomics analysis to resolve a taxonomic question utilises considerably more data than those based on multigene phylogenies. However, despite the larger body of data, this approach failed to resolve the issue as to whether the isolates of *Ceratocystis* residing in the LAC group are taxonomically robust species. All researchers in the field agree that *C. platani* is a species distinct from *C. fimbriata* but these two species have also been reported to be interfertile [12]. In addition, Fourie et al. [47] reported significant transmission ratio distortion in a cross between *C. manginecans* and *C. fimbriata*, providing evidence that these two species or populations have been reproductively isolated for some time. Results of the present study show there is greater separation between *C. platani* than between *C. manginecans*, *C. fimbriata* and *C. eucalypticola*. It is also clear that within a well-defined species such as *C. albifundus* there is more obvious recombination than is evident than in the LAC.

## Conclusions

Phylogenomic analyses of representative species in Ceratocystidaceae revealed widespread incongruence among single gene trees. Our analyses showed evolutionary patterns consistent with those of introgressive hybridization although the similar effects of incomplete lineage sorting could not be completely ruled out and is subject to further studies. The concatenated dataset was able to resolve some of the incongruence suggesting a phylogenomic approach could be necessary for the phylogeny of these species. As such, we recommend that future taxonomic analyses of species in the Ceratocystidaceae should apply a phylogenomic approach incorporating larger populations sampled from different hosts and geographical regions to maximise genetic variability.

## Methods

### Isolates and genome information

Seventeen isolates representing 10 species across three genera of the Ceratocystidaceae [7], were chosen for this study (Table 1). The collection included 14 isolates representing seven species of *Ceratocystis*, two representative isolates of *Endoconidiophora* and a single *Davidsoniella* isolate. These isolates were selected based on the fact that whole genome sequence data were available for them [40, 48–54]. The sequences were downloaded from the National Centre for Biotechnology Information database (NCBI; http://www.ncbi.nlm.nih.gov/).

### Ortholog selection using BUSCO analysis

Single copy orthologs for phylogenomic analysis were identified using the Benchmarking Universal Single-Copy Orthologs (BUSCOs) tool (BUSCO v1.1b1). The BUSCO genomics tool performs an assessment of genome assembly completeness by quantifying the percentage of core genes present from a specific lineage (Simão et al. [35]). BUSCO was run in genome mode with the fungal lineage dataset. The remaining parameters focused on optimal training during the AUGUSTUS annotation step (*−-long*), with the gene models of *Fusarium graminearum* being specified*. Fusarium* is the phylogenetically closest fungal genus for which these models were available. The shared, complete, single copy BUSCOs were extracted into fasta files using BEDtools v2.26.0 [55, 56].

### Phylogenetic analyses

Characterization of the shared BUSCO genes across the Ceratocystidaceae was performed using Blast2GO’s default pipeline [57–59] to identify functions possibly linked to patterns observed in the phylogenomic analysis. Multiple sequence alignments (MSAs) of the BUSCO nucleotide sequences were generated using MAFFT v7 [60, 61] and curated using Gblocks v0.91b [62] to remove gaps and to rectify misaligned regions. The curated MSAs were concatenated using FASconCAT-G [63, 64], and a Maximum Likelihood (ML) species phylogenetic tree constructed using RAxML [65, 66] with 1000 bootstrap replicates performed. Additionally, to confirm the ML species tree topology, a Bayesian species tree was generated with MRBAYES with one million generations [67]. Individual ML gene trees were generated for each BUSCO alignment using RAxML with 100 bootstrap replicates. Substitution models for the nucleotide ML gene trees (individual gene trees and species tree) used the GTR model as a default parameter. DensiTree [68] was used to visualise the incongruent phylogenies by drawing all topologies in a tree set on the same image using transparency to show conflict. MetaTree was used to compare and visualize multiple alternative tree topologies [69]. The gene trees were first transformed in Figtree v1.4.2 (tree.bio.ed.ac.uk/software/figtree/) to make all branch lengths proportional, ensuring overlap for tree topology comparison in DensiTree. Compare2trees was used for comparisons between two alternative topologies [36].

## Supplementary information

**Additional file 1:Figure S1.** Pie chart summarizing the biological processes of the shared BUSCO genes in the analysed Ceratocystidaceae. The numbers in brackets represent the number of GO (Gene Ontology) annotations.

**Additional file 2:Figure S2.** (A) MetaTree analysis of 1121 amino acid ML gene trees. The amino acid ML gene trees clustered showing major star-like radiation indicating a lack of phylogenetic resolution. (B) MetaTree analysis of 1121 nucleotide ML gene trees. The highlighted cluster shows the consensus trees of the *C. fimbriata*, *C. manginecans* and *C. eucalypticola* clade representing approximately 72% of all ML gene trees. The remaining clusters are supported by small numbers of the remaining ML gene trees.

**Additional file 3:Figure S3.** The three main consensus topologies of the DensiTree analysis of the 1069 nucleotide ML gene trees including all 17 Ceratocystidaceae genomes analysed. Topology 1 representing 17% of all gene trees is coloured in blue, topology 2 representing 16.5% of all ML gene trees is coloured in red, and topology 3 representing 16% of all ML gene trees is coloured in green. See Table 1 in main article for full species names.

**Additional file 4:Figure S4.** A Bayesian species tree for the Ceratocystidaceae species analysed. The GTR model with gamma distribution and one million generations in two runs were used. A burnin of 25% was applied when summarising the trees. All other parameters were set to default. The average standard deviation of tree splits was zero and the species tree nodes were absolutely supported with posterior probabilities of 1.

## Data Availability

The Whole Genome Shotgun projects have been deposited at DDBJ/EMBL/GenBank under accession numbers listed in Table 1 and are available in the National Centre for Biotechnology Information (NCBI) GenBank database (https://www.ncbi.nlm.nih.gov/genbank/). Additional results supporting findings presented in this study are provided in the additional information section.

## Abbreviations

aa: amino acid
AAC: Asian-Australian Clade
AFC: African Clade
BSC: Biological Species Concept
BUSCO: Benchmarking Universal Single-Copy Orthologs
GCPSR: Genealogical Concordance Phylogenetic Species Recognition
ITS: Internal Transcribed Spacer of ribosomal RNA genes
LAC: Latin American Clade
Mbp: Mega base pair
ML: Maximum Likelihood
MSC: Morphological Species Concept
NAC: North American Clade
PSC: Phylogenetic Species Concept
