# Supplementary figures and images for: Phylogenomic incongruence in Ceratocystis: a clue to speciation?
Source: BMC Genomics. 2020 May 14;21:362. doi: 10.1186/s12864-020-6772-0 (PMC7222570; doi:10.1186/s12864-020-6772-0)

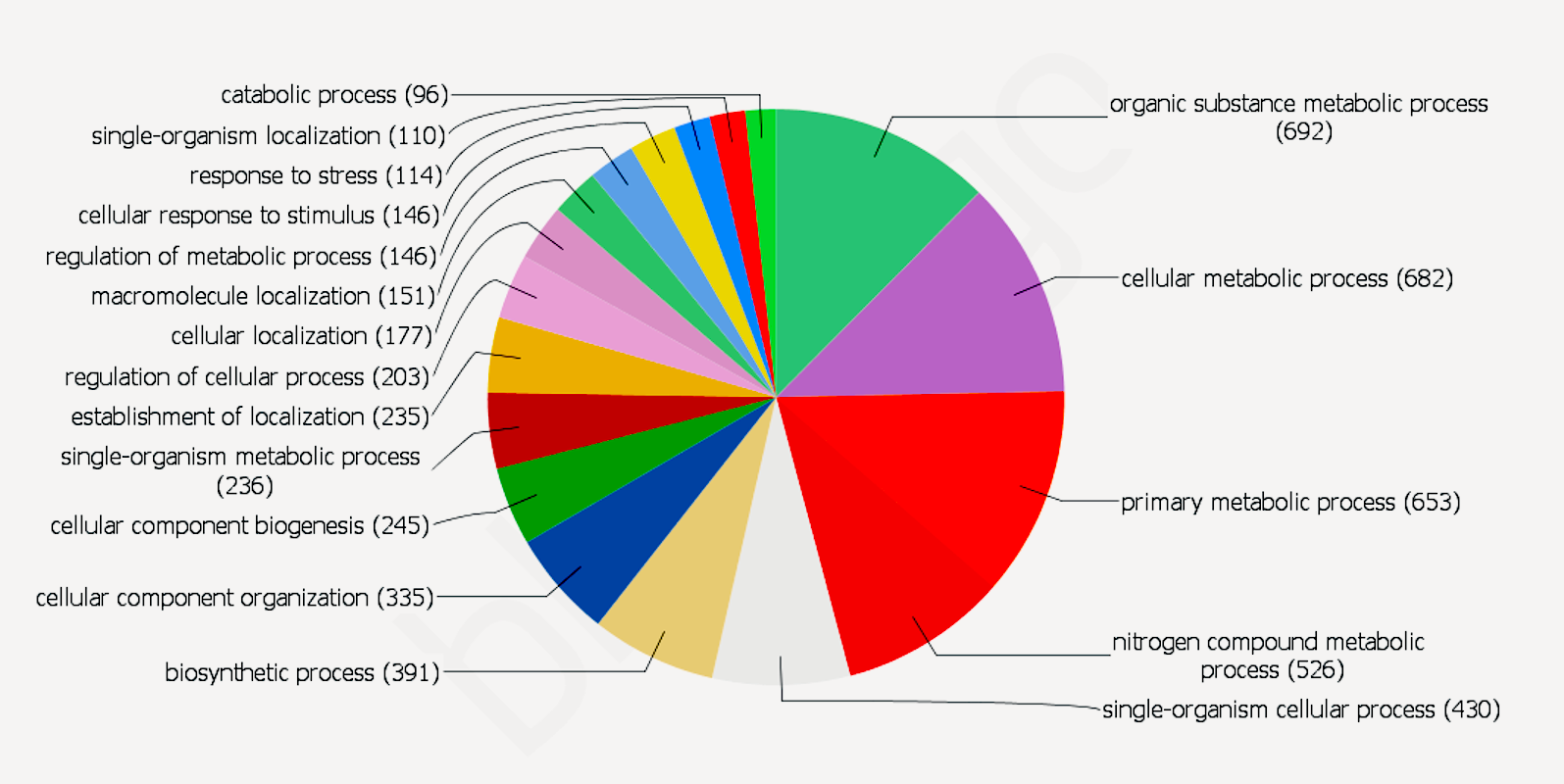

Supplement: Supplementary file 1 — Additional file 1:Figure S1. Pie chart summarizing the biological processes of the shared BUSCO genes in the analysed Ceratocystidaceae. The numbers in brackets represent the number of GO (Gene Ontology) annotations. [file 12864_2020_6772_MOESM1_ESM.tiff]

**A**

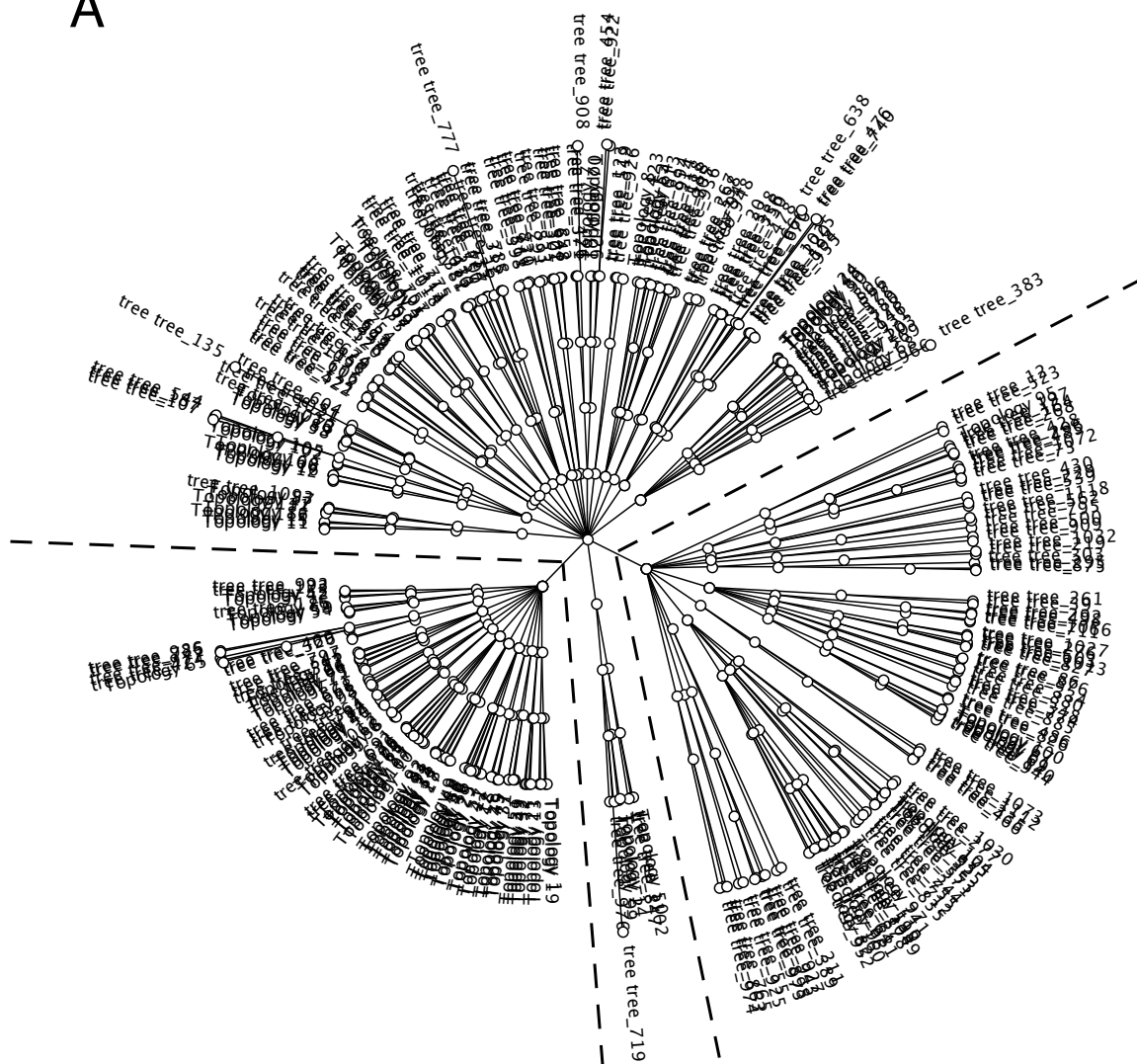

Supplement: Supplementary file 2 — Additional file 2:Figure S2. (A) MetaTree analysis of 1121 amino acid ML gene trees. The amino acid ML gene trees clustered showing major star-like radiation indicating a lack of phylogenetic resolution. (B) MetaTree analysis of 1121 nucleotide ML gene trees. The highlighted cluster shows the consensus trees of the C. fimbriata, C. manginecans and C. eucalypticola clade representing approximately 72% of all ML gene trees. The remaining clusters are supported by small numbers of the remaining ML gene trees. [file 12864_2020_6772_MOESM2_ESM.zip › Figure-S2A.pdf]

# B

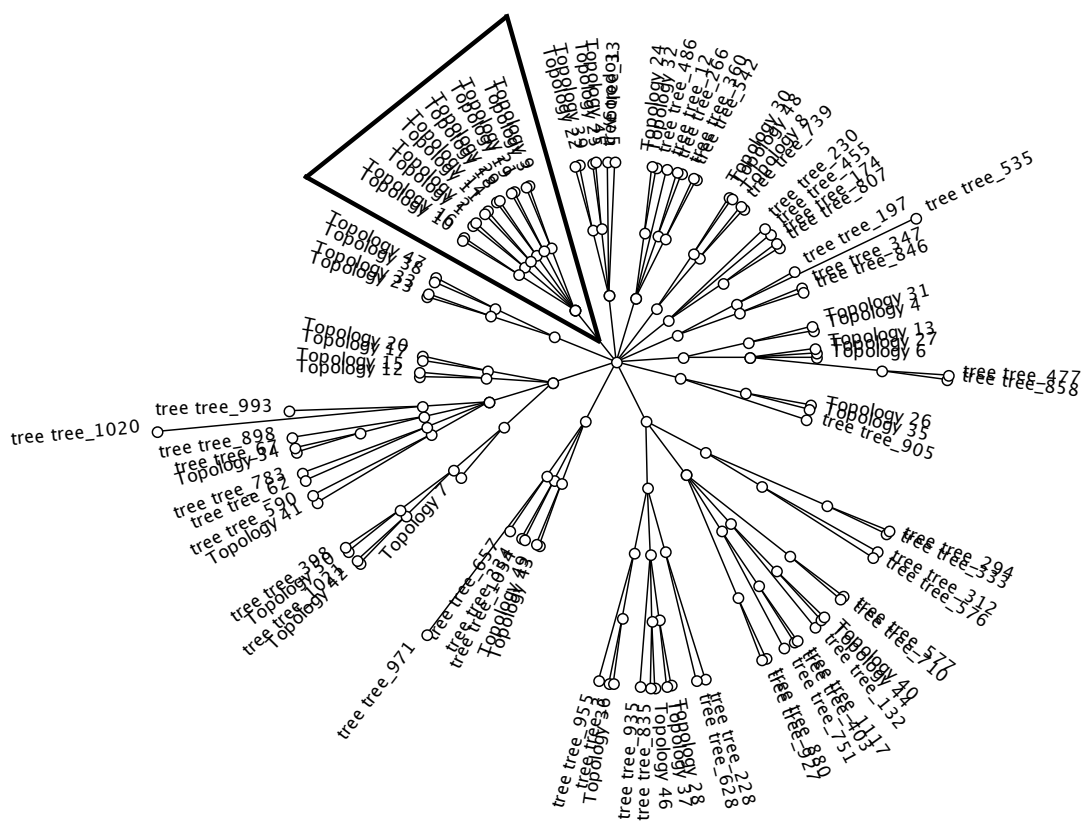

Supplement: Supplementary file 2 — Additional file 2:Figure S2. (A) MetaTree analysis of 1121 amino acid ML gene trees. The amino acid ML gene trees clustered showing major star-like radiation indicating a lack of phylogenetic resolution. (B) MetaTree analysis of 1121 nucleotide ML gene trees. The highlighted cluster shows the consensus trees of the C. fimbriata, C. manginecans and C. eucalypticola clade representing approximately 72% of all ML gene trees. The remaining clusters are supported by small numbers of the remaining ML gene trees. [file 12864_2020_6772_MOESM2_ESM.zip › Figure-S2B.pdf]

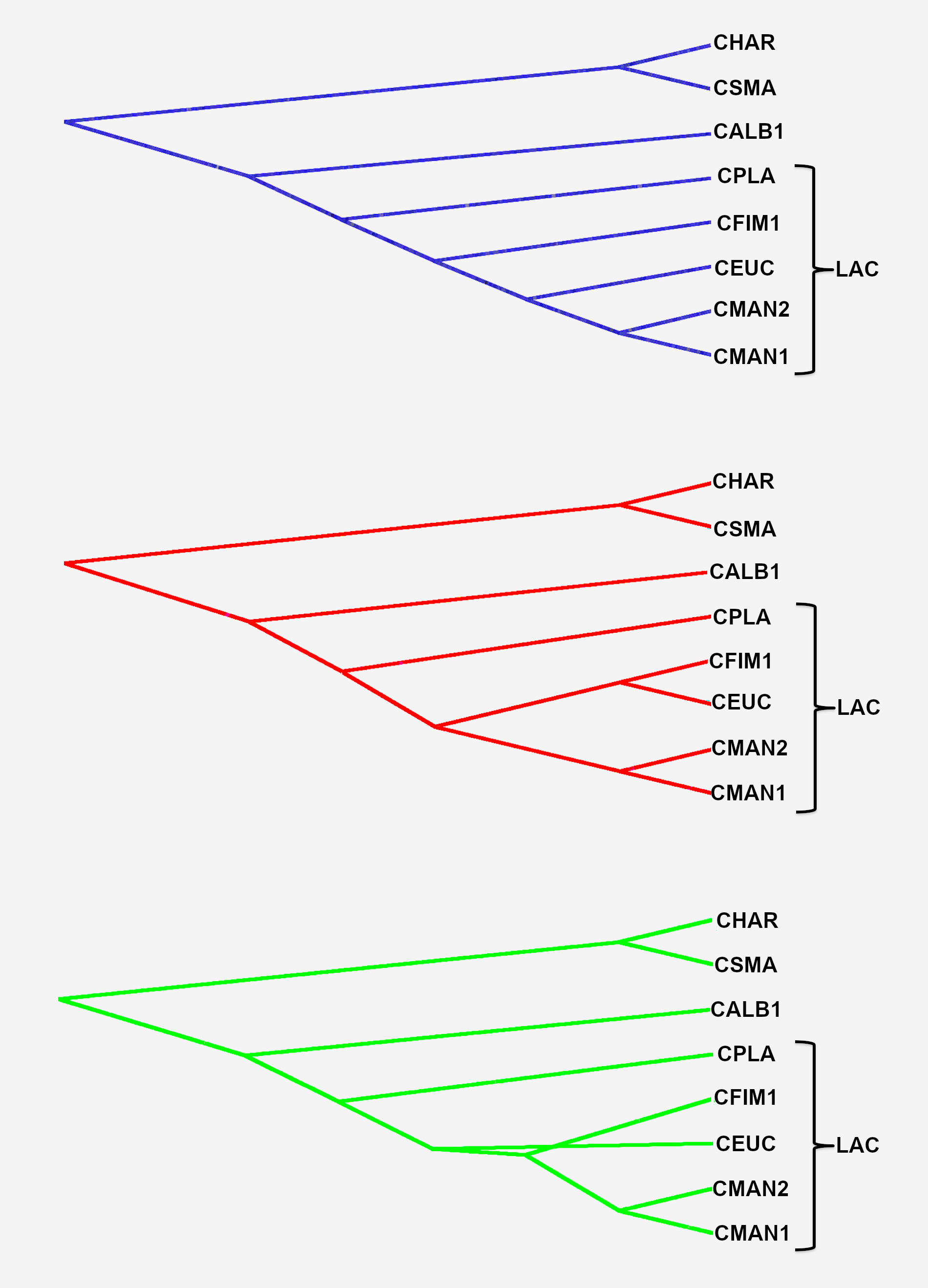

Supplement: Supplementary file 3 — Additional file 3:Figure S3. The three main consensus topologies of the DensiTree analysis of the 1069 nucleotide ML gene trees including all 17 Ceratocystidaceae genomes analysed. Topology 1 representing 17% of all gene trees is coloured in blue, topology 2 representing 16.5% of all ML gene trees is coloured in red, and topology 3 representing 16% of all ML gene trees is coloured in green. See Table 1 in main article for full species names. [file 12864_2020_6772_MOESM3_ESM.tiff]

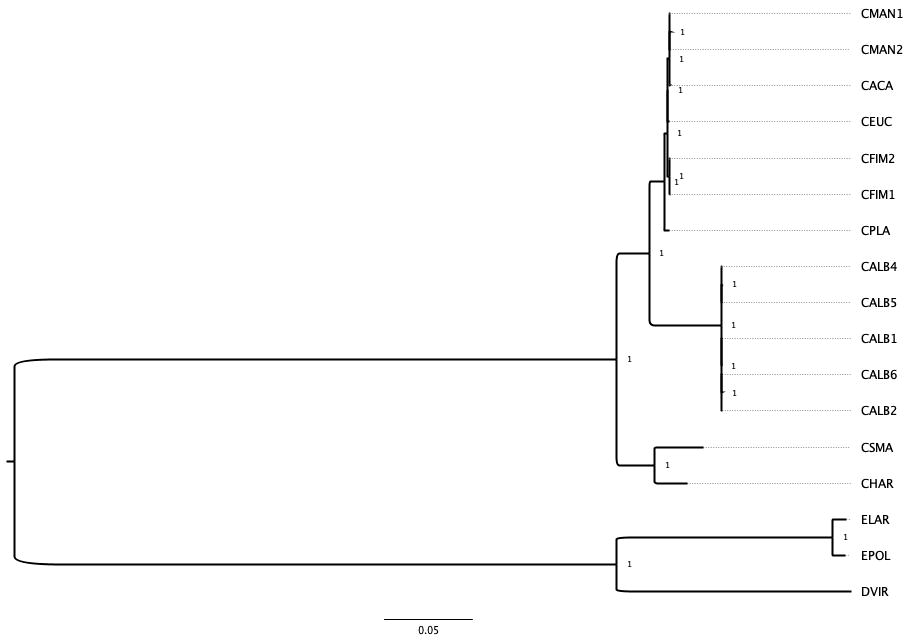

Supplement: Supplementary file 4 — Additional file 4:Figure S4. A Bayesian species tree for the Ceratocystidaceae species analysed. The GTR model with gamma distribution and one million generations in two runs were used. A burnin of 25% was applied when summarising the trees. All other parameters were set to default. The average standard deviation of tree splits was zero and the species tree nodes were absolutely supported with posterior probabilities of 1. [file 12864_2020_6772_MOESM4_ESM.tiff]
